# Optimal profile limits for maternal mortality rate (MMR) in South Sudan

**DOI:** 10.1186/s12884-018-1892-0

**Published:** 2018-07-03

**Authors:** Gabriel Makuei, Mali Abdollahian, Kaye Marion

**Affiliations:** School of Science (Mathematical and Geospatial Sciences), College of Science, Engineering, and Health, RMIT University, GPO BOX 2476, Melbourne, VIC 3001 Australia

**Keywords:** Profile limits, Optimisation, Maternal mortality rate, Ln multi-regression, South Sudan

## Abstract

**Background:**

Reducing Maternal Mortality Rate (MMR) is considered by the international community as one of the eight Millennium Development Goals. Based on previous studies, Skilled Assistant at Birth (SAB), General Fertility Rate (GFR) and Gross Domestic Product (GDP) have been identified as the most significant predictors of MMR in South Sudan.

This paper aims for the first time to develop profile limits for the MMR in terms of significant predictors SAB, GFR, and GDP. The paper provides the optimal values of SAB and GFR for a given MMR level.

**Methods:**

Logarithmic multi- regression model is used to model MMR in terms of SAB, GFR and GDP. Data from 1986 to 2015 collected from Juba Teaching Hospital was used to develop the model for predicting MMR. Optimization procedures are deployed to attain the optimal level of SAB and GFR for a given MMR level.

MATLAB was used to conduct the optimization procedures. The optimized values were then used to develop lower and upper profile limits for yearly MMR, SAB and GFR.

**Results:**

The statistical analysis shows that increasing SAB by 1.22% per year would decrease MMR by 1.4% (95% CI (0.4–5%)) decreasing GFR by 1.22% per year would decrease MMR by 1.8% (95% CI (0.5–6.26%)).

The results also indicate that to achieve the UN recommended MMR levels of minimum 70 and maximum 140 by 2030, the government should simultaneously reduce GFR from the current value of 175 to 97 and 75, increase SAB from the current value of 19 to 50 and 76.

**Conclusions:**

This study for the first time has deployed optimization procedures to develop lower and upper yearly profile limits for maternal mortality rate targeting the UN recommended lower and upper MMR levels by 2030. The MMR profile limits have been accompanied by the profile limits for optimal yearly values of SAB and GFR levels. Having the optimal level of predictors that significantly influence the maternal mortality rate can effectively aid the government and international organizations to make informed evidence-based decisions on resources allocation and intervention plans to reduce the risk of maternal death.

## Background

Improving maternal health and reducing related mortality has been the key concern of the international community as one of the Eight Millennium Development Goals (MDG) [1]. Maternal health is a major global development challenge particularly in Africa which accounts for about half of the world’s maternal deaths, with little or no progress towards reduction of maternal mortality. Factors associated with maternal mortality in sub-Saharan Africa (SSA) include prenatal care coverage and skilled attendance at delivery. Kruk, et al., (2010) investigated the impact of the community perceptions on the quality of care provided by the local health system on pregnant wom**e**n’s decision**s** to deliver in a clinic. They suggest that improving the quality of care at first level clinics may assist the efforts to increase facility delivery in sub-Saharan Africa [2]. However, there are other contributing factors including socio-economic factors, macro-economic factors and physiological factors. South Sudan has about 1147 health care facilities that function to serve population of around 13 million. Out of these facilities, only 37 are hospitals. Ill-equipped buildings with poor hygiene are the common feature of these primary health care units. Chronic shortage of health care professional staff at all levels is demonstrated by 1.5 doctors and two nurses per 100,000 people (National Bureau of Statistic [3]’ Report, (2013, 2014, 2015), and World Health Organization, (2014) [4]. All the above factors affect the total health care system and in particular the high maternal mortality rate problem.

The maternal mortality rate (MMR) in South Sudan is one of the highest in the world [3, 5, 6]. The risk of a pregnant woman dying is as high as one in seven. In Africa overall the risk of a pregnant woman dying is one in 16, in contrast with Asia (1 in 105), Europe (1 in 1895) and in North America (1 in 3750), [4, 7, 8], [9]. Thus, there is an urgent need for evidence-based intervention to significantly reduce mortality rate in South Sudan.

The data released by WHO, UNICEF, UNFPA, World Bank and the United Nations Division, [4] shows that even though the maternal mortality rate in South Sudan has decreased from 1000 to 730 per 100,000 live births between 2005 to 2015, it is still one of the highest in the world. Combining this with the high fertility rates in the country gives the probability of an average reproductive South Sudanese woman (12–49 years of age) dying during pregnancy to be 14.3% [10]. Therefore, understanding the trends in MMR and factors significant to MMR would be vital for developing health care systems that can ensure safe delivery.

The World Health Organization and USAID (2015) have recommended that all countries with high MMR level should aim to reduce MMR to the minimum of 70 and the maximum of 140 by 2030 [7].

This paper only investigates the impact of the most influential economic factors on the non-HIV MMR in South Sudan [11]. The authors have identified the most economical influential factors for MMR in South Sudan [10]: The Skilled Attendants at Births (SAB), the General Fertility Rate (GFR) and the Gross Domestic Product (GDP). The MMR is expressed as the annual number of maternal deaths per 100,000 live births. The SAB is the annual percentage of Skilled Attendants at Birth; the GFR is expressed as the annual percentage of live births per 1000 women of reproductive age (ages of 12 to 49 years) in a population. The GDP is expressed in US Dollars (USD). The relevant data for this research was collected for the period from January 1986 to October 2015 from Juba Teaching Hospital **(**JTH) which is one of the major health care referral centres in South Sudan. The hospital is a 580-bed facility located in the capital city. Additional data was collected from other reliable sources such as: Reproductive Health Department from Ministry of Health (MoH), National Bureau of Statistics NBS Report [12], the South Sudan 2009 National Baseline Household Survey Report, South Sudan Household Health Survey [13], Census of Population and Housing [14], and United Nations’ organizations and their partners (e.g. WHO, UNAID, UNICEF, UNDP).

The findings show that Generals Fertility Rate (GFR) is the most influential factor in increasing the maternal death (MDs) followed by skilled attendant at birth (SAB) and the Gross Domestic Product (GDP). MMR decreases when SAB increases and the GFR decreases.

In their comprehensive discussions regarding strategies to reduce MMR, the Campbell, Graham, and Lancet Maternal Survival Series Steering Group [15] stressed the intra-partum care, which includes different types of skilled assistants at birth. Use of contraception to reduce the fertility rate is also mentioned though considered less important. However, the viability of both methods is positive for South Sudan.

According to the data from Juba Teaching Hospital (JTH) any efforts to increase SAB through increasing the number of trained attendants either directly or indirectly would affect the GFR and the GDP. According to the current statistics, only 20% births are attended by skilled assistants [3]. This indicates the need to increase the SAB and decrease the GFR levels. For more rapid impact on MMR, however, the programme of training birth attendants at village and community levels needs to be accelerated and this involves considerable funding.

Currently, the GFR in South Sudan is very high at around five children per mother (6.9 in 2014 cited by the Population Reference Bureau, 2016) [16] which is double the global average of 2.5%. Decrease in GFR has a much longer term effect in reducing MMR, however, contraceptive use has only reached 4% of the population compared to 62% globally [16]. Consequently, there is scope for improvement in this respect; yet this also involves substantial funding. However, acceleration in reducing GFR involves substantial funding to provide improved and affordable health care access to pregnant women. Although increase in GDP would increase MMR, because of the cultural practice of polygamy, the country needs economic growth as a fundamental requirement to provide improved and affordable health care access to pregnant women.

The only likely strategy in the current environment is to increase SAB while decreasing GFR in order to reduce MMR. The importance of attending to socio-economic factors to reduce MMR was highlighted in recent Indian research by Rai, and Tulchinsky (2015) [17]. Decreased fertility rate will decrease the number of births and thus reduce the number of SAB required. Consequently large scale effort to use birth control methods and increase trained birth attendants at village and community levels needs to be implemented.

In this paper we use the logarithmic multi-regression model suggested by Makuei et al. [10] to estimate MMR based on SAB, GFR and GDP. The mathematical optimization is then used to obtain the optimal values of SAB and GFR (when GDP is kept constant) for a given level of MMR. This study for the first time aims to develop yearly lower and upper profile limits for the MMR expressed in terms of significant predicators SAB, GFR and GDP using real data collected from the resources outlined above. The MMR profile limits are then accompanied by yearly optimal profile limits of SAB and GFR to achieve the recommended UN MMR levels by 2030. The optimal profile limits provide a quantitative guide-line for the government and partners in terms of yearly SAB and GFR targets in order to reduce MMR to the level recommended by the UN agencies [4].

Based on the previous study, Log Linear Regression can model maternal deaths more accurately compared with Poisson regression [10]. Thus, Log Linear Regression model has been developed and is used for optimization purpose. A reliable model that can estimate the maternal deaths and the optimized values of its corresponding predictors will assist the government to make an inform decision on resource allocation and lacking resources in order to reduce the domestic MMR.

The results of our analysis show that increasing SAB rapidly to the highest level is possible. Under this condition, a slow increase in GFR will not increase MMR too much above the UN maximum recommended level of 140. On the other hand, considering that the GFR in South Sudan is already at almost the highest level, it is more likely that it will decrease with increased education, income levels and awareness.

## Methods

This section outlines the data collection tasks, prediction model for MMR, mathematical optimization and development of linear profile limits for MMR in terms of its significant predictors.

### Data collection

The research has used 30 years of data (1986–2015) to carry out the statistical analysis. The data was collected from the Department of Statistics at the Juba Teaching Hospital (JTH), Reproductive Health Department from Ministry of Health (MoH) [3] National Bureau of Statistics NBS Report [12], the South Sudan 2009 National Baseline Household Survey Report, South Sudan Household Health Survey [13], Census of Population and Housing [14], and the United Nations’ Organizations and their partners (e.g. WHO, UNAID, UNICEF, UNDP).

The yearly data included the number of non-HIV+/AIDS maternal deaths, yearly SAB, GDP and yearly GFR. The data on GDP was mainly obtained from National Bureau of Statistics (NBS) Yearly’ Report, World Health Organization (WHO), UNICEF, the World Bank and the United Nations Population Division [12].

### Prediction model for MMR

Several models for predicting MMR based on different predictors were developed by Makuei, et al. [10]. We used randomly selected two third of the Yearly Data to build the models. The models were then used to predict the remaining ten years’ data. The mean errors and the standard error of the mean (SE Mean) were used to compare the efficacy of the models. The analysis was carried out using Microsoft Excel, R and Minitab version 17 statistical soft-ware.

The following two models were the predictive models best describing MMR (based on their respective mean error and SE Mean):

Log Regression Equation, *R*^*2*^ *= 77.11%*

1 $$ Log\ \left( Non- HIV/ AIDS\right)=\hbox{-} 20.8\hbox{-} 8.30\  Log\ (SAB)+ 8.10\  Log\ (GFR)+ 5.12\  Log\ (GDP) $$

Poisson Regression Equation, *R*^*2*^ *= 79.75%.*

Non-HIV+/AIDS MDs Rate Per 1000 = exp.(Y′)

2 $$ {Y}^{\hbox{'}}= 4.227- 0.3819\  SAB+ 0.03237\  GFR+ 0.002902\  GDP $$

Since mean errors and SE Mean for the Log Linear Regression is much less than Poisson Regression, we can conclude that Log linear regression outperforms Poisson regression in predicting the MMR for South Sudan.

In this paper we deployed Ln-regression and used 30 years of data to develop the following optimal prediction model for MMR.

3 $$ \mathrm{Ln}\ \left(\mathrm{MMR}\right)=-10-1.73\ast \mathrm{Ln}\ \left(\mathrm{SAB}\right)+2.83\ast \mathrm{Ln}\ \left(\mathrm{GFR}\right)+0.943\ast \mathrm{Ln}\ \left(\mathrm{GDP}\right) $$

Equation (3) indicates that one unit change in Ln (SAB) will decrease Ln (MMR) by 1.73 units while one unit change in Ln (GFR) and Ln (GDP) will increase Ln (MMR) by 2.83 and 0.943 respectively. As the relationships are logarithmic, the effect on actual values of MMR, in terms of maternal death per 100,000 live births, will be several times higher.

Compared to the decrease in MMR which can be brought about by increasing Ln (SAB), the increasing effect of Ln (GFR) on Ln (MMR) is much higher (1.64 times) than that of Ln (SAB). Meanwhile, the effect that one unit change in Ln (GDP) has on Ln (MMR) is (0.55 times) less than that of Ln (SAB).

This result on GDP is aligned with the finding by the authors in [32] who investigated the relationship between MMR and GDP in 79 developing countries and concluded that per capital GDP was one of the most significant predictor (− 0.83) for MMR. Similarly in China, Feng XL et al. observed that log (GDP) per capita was a determinant of crude MMR with the adjusted rate ratio of 0.85–0.86 compared to a crude ratio of 0.66 [18] . In their study Du et al. noted that reduction of MMR over the period of 1996–2009 in Guizhou province of China was negatively related to GDP [19] . In our study, due to lack of the yearly GDP data in South Sudan, the GDP value was held constant at the average GDP over the period of 1986–2015 (Ln (GDP) =7.480, or GDP = 1772).

Optimization is often used to optimize and observe optimal values, patterns and structures in data over time. In this analysis we used MATLAB, Excel Solver, R, and Minitab 17. They have been used to conduct optimization procedures and obtain optimal values for the predictors SAB and GFR for a given value of MMR. The optimized values were then used to develop and plot lower and upper profile limits for the MMR, SAB and GFR to achieve the UN recommended lower and upper MMR limits of between 70 and 140.

### Mathematical optimization

In mathematics, computer science, and operation research, mathematical optimization is the selection of a best value from some set of available alternatives. An optimization problem consists of minimizing or maximizing a real function systematically by choosing input values for its individual variables from within an allowed domain. In addition to that, optimization includes.

Finding the best available values of some objective function given a defined domain or input including a variety of various types of constrains.

### Optimization using solver package and MATLAB

**Fig. 1:**
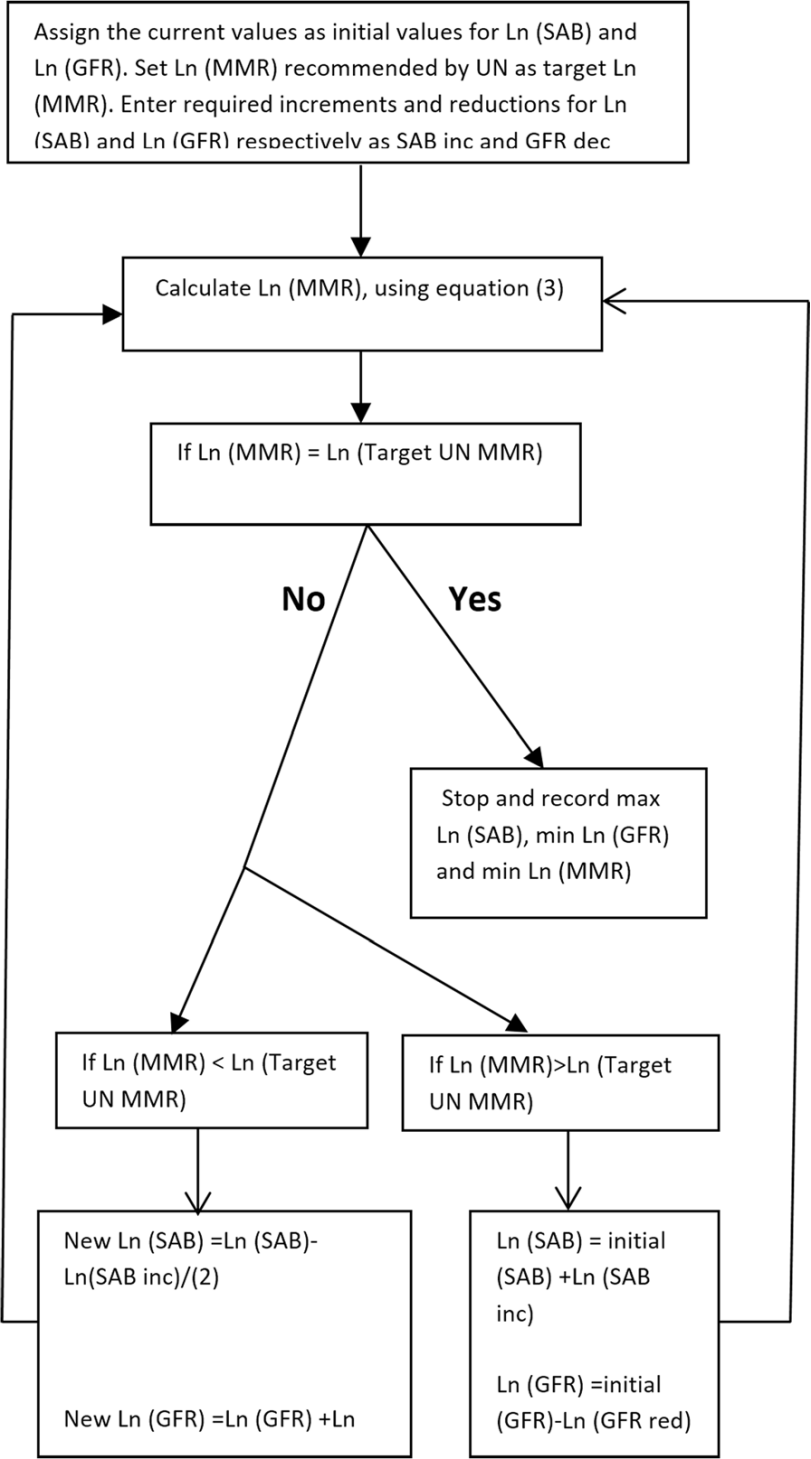
Calculation optimal max Ln (SAB) and min Ln (GFR) values for a given MMR level

Solver is part of a collection of commands to determine the minimum or maximum value of one cell by changing the values of other cells. With Solver, an optimal (minima or maxima) value could be found for the objective cell, subject to constraints or limits on the values of the predictors. In this study Ln (SAB) and Ln (GFR) were optimized for given values of Ln (MMR) while keeping Ln (GDP) constant at 7.480 or GDP = 1772 which is the average of Ln (GDP) over the period that data were collected. The results from solver are then confirmed with the results of the algorithm developed in MATLAB. Optimization procedures to attain optimal max Ln (SAB) and min Ln (GFR) values for a given Ln (MMR) level is outlined in the algorithm presented in Fig. 1 below.

### Linear profile limits

Profile monitoring systems assist and help to identify factors related to an observed phenomenon, assess the effect of changing any factor/s on the event and predict the behaviour of the phenomenon under different situations. In many situations the quality and performance of a process may be better characterized and summarized by relationship between the response (dependent) variable and one or more explanatory (independent) variables referred to as profile [20].

The general parametric linear profile model relating the explanatory variables X_1i_, X_2i,_ X_3i_ ..., X_pi_ to the response Y_ij_, is presented by.

$$ {\mathrm{Y}}_{\mathrm{ij}}={\mathrm{A}}_{0\mathrm{j}}+{\mathrm{A}}_{1\mathrm{j}}{\mathrm{X}}_{1\mathrm{i}}+.\dots, +{\mathrm{A}}_{\mathrm{pj}}+{\upvarepsilon}_{\mathrm{ij}},\mathrm{i}=1,2,3,\dots, \mathrm{n},\kern0.5em \mathrm{j}=1,2,3,\dots, \mathrm{k} $$

where A_1j_ (l = 0,1,2, ...., p) is the regression coefficient. The pair observation (X_li_, Y_ij_) is obtained in the jth random sample, where X_li_ is the ith design point (i = 1,2,3, ..., n) for the lth explanatory variable (l = 1.2,3, ..., p ). It is assumed that the errors ɛ_ijs_ are independent, identically distributed (i.i. d.) variables with mean zero and variance σ^2^_j_, when the process is in control.

Profile monitoring is used to understand and to check the stability of this relationship over time [21].

Recently many practitioners and researchers have used profile monitoring as a new sub-area of statistical process control exploring the application of profile monitoring in different disciplines and in real life [22–24]. The application of profile monitoring is often focussed on processes with multiple quality characteristics and has also been extended to detect clusters of disease incident and used in public health surveillance [25–32].

In this study, profile monitoring will be used to monitor maternal mortality rate (MMR) in South Sudan and assess its variation influenced by SAB and GFR.

### Development of profile limits

In this paper, MATLAB, Minitab, R and Excel Solver are used to obtain optimal values of Ln (SAB) and Ln (GFR) for a given value of Ln (MMR) while keeping Ln (GDP) constants at 7.480 (GDP = 1772) which is the average of Ln (GDP) over the period that the data were collected.

Furthermore, to generate the lower and upper profile control limits for Ln (MMR), the proposed predictive models presented in eq. (3) and the target minimum and maximum levels of MMR proposed by the UN agencies; MMR = 70 and MMR = 140 have been used. It was recommended that these limits should be achieved by 2030. The current MMR in South Sudan is about 730 deaths per 100,000.

The statistical analysis shows that increasing SAB by 1.22% per year would decrease MMR by 1.4% (95% CI (0.4–5%)) while decreasing GFR by 1.22% per year would decrease MMR by 1.8% (95% CI (0.5–6.26%)).

The following steps were taken to generate the lower and upper profile limits for yearly target values of SAB and GFR in order to reduce MMR to the target minimum and maximum levels recommended by UN agencies.

**Fig. 2:**
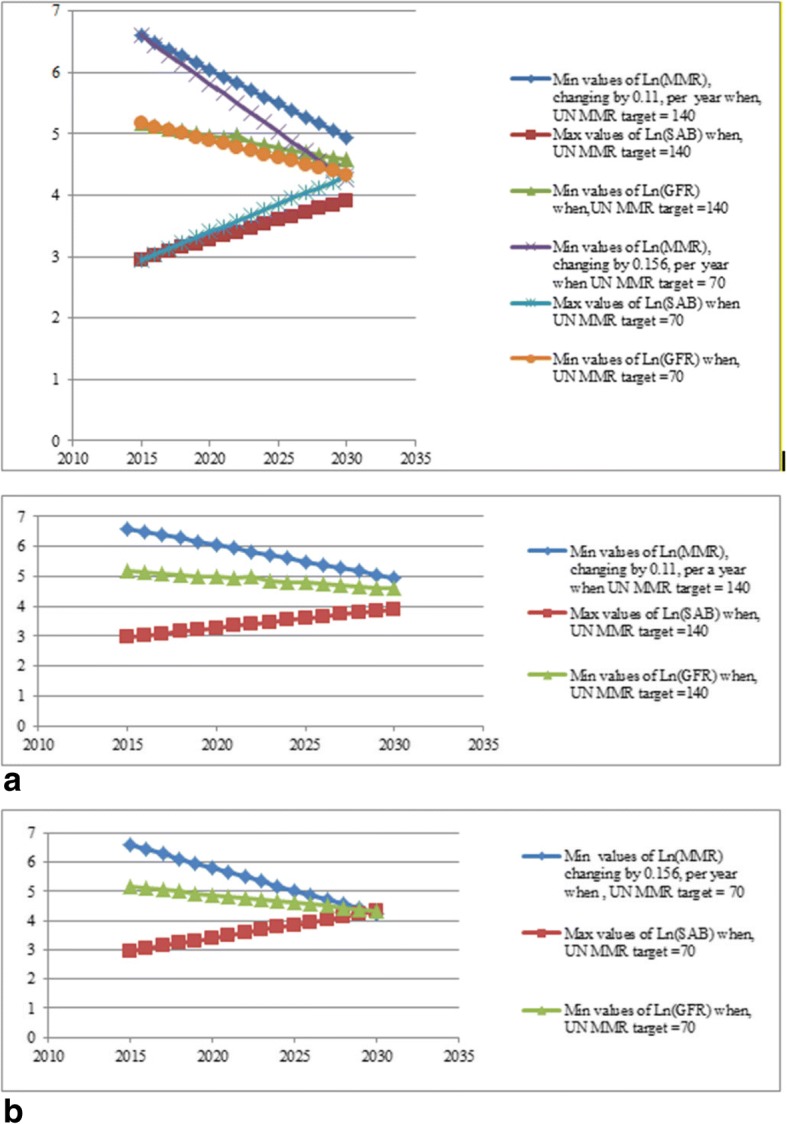
Lower and upper profile limits for Ln (MMR), Ln (SAB) and Ln (GFR). **a** Profile limits for Ln (MMR), Ln (SAB) and LN (GFR). The target MMR for 2030 is 140. **b** Profile limits for Ln (MMR), Ln (SAB) and LN (GFR). The target MMR for 2030 is 70

**Fig. 3:**
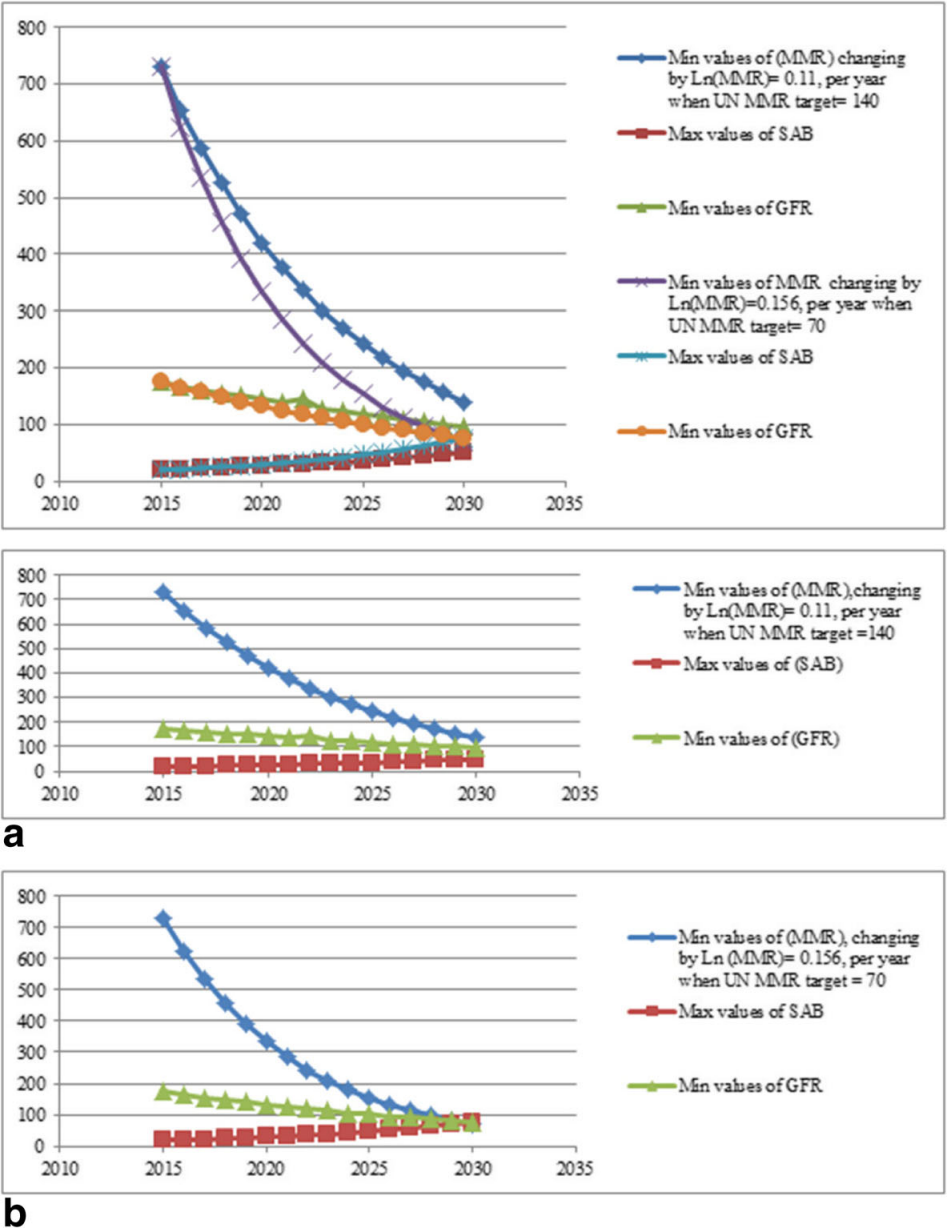
The lower and upper profile limits for MMR, SAB and GFR. Figure 3a: Profile limits for the numerical values of MMR, SAB and GFR. The target MMR for 2030 is 140. Fig. 3b: Profile limits for the numerical values of MMR, SAB and GFR. The target MMR for 2030 is 70

**Fig. 4:**
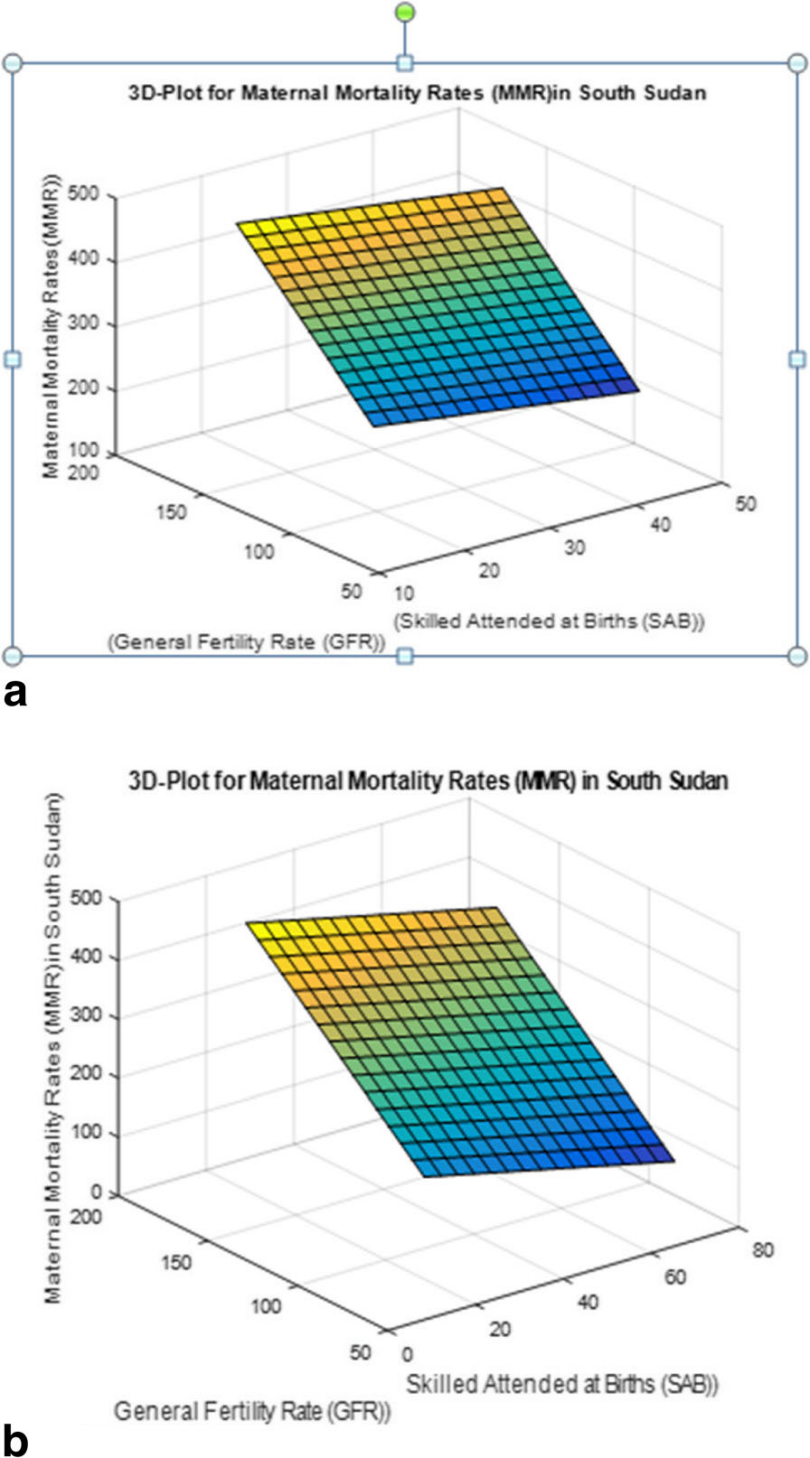
**a** Three dimensional Surface Plot of MMR values vs SAB and GFR for target MMR 140. **b** Three dimensional Surface Plot of MMR values vs SAB and GFR for target MMR 70

- **Step 1** To achieve MMR = 140 (the maximum recommended by the UN) from the current value of 730 by 2030, the government should reduce MMR by (approximately) 39 deaths per year (or Ln (MMR) by 0.11 per year). Therefore, the optimization program was deployed to obtain the optimal sets of Ln (SAB) and Ln (GFR) for a given Ln (MMR) with the starting value of Ln (730). The Ln (MMR) was then reduced by 0.11, year by year. The results in terms of Ln function and numerical values are presented in Tables 1 and 2. The profile limits are presented in Figs. 1, 2, 3 and 4. It should be noted that the constraint on Ln (SAB) is that, it should be greater than the existing maximum (Ln (SAB) = 3.178), as our aim is to increase SAB year by year. While the constraint on Ln (GFR) was to be smaller than the existing current minimum value of 5.024, its value should be further decreased. The results presented in the first 3 columns of Table 2 show that to decrease MMR from 730 to 140 by the year 2030, the government should increase SAB from the current value of 19 to 50 while the value of GFR should be decreased from the current value of 175 to 97. The five years break-up values are highlighted in Table 2. Thus, for the year 2020, South Sudan should target to have MMR decreased from the current value of 730 to 421 by simultaneously increasing SAB from 19 to 26 and deceasing GFR from 175 to 144. By the year 2025 the country should target to have MMR declined from the present value of 730 to 243 by simultaneously increasing SAB from 19 to 36 and decreasing GFR from 175 to 118. Moreover, by the year 2030, the government and stakeholders should target to have MMR decreased from the current value of 730 to 140 by increasing SAB from 19 to 50 and decreasing GFR from 175 to 97.
- **Step 2** To attain MMR = 70 (the minimum recommended by the UN) from the current value of 730 by 2030, step one was followed except that the target value was changed from 140 to 70 and the decrease in Ln (MMR) was 0.156 per year. The optimization results in terms of Ln function and numerical values are presented in Tables 1 and 2. The last three columns of Table 2 show that to achieve MMR of 70 by the year 2030, the authorities in South Sudan should reduce GFR from 175 to 75 while increasing SAB from the current value of 19 to 76. The target statistics for 2020 would be MMR = 334 with SAB being increased to 30 and GFR reduced to 133. By the year 2025 the government and partners should target to have MMR decreased from the present value of 730 to 153, by simultaneously increasing SAB from 19 to 47 and decreasing GFR from 175 to 101. Therefore, developing health policies that target MMR, the SAB and GFR profile limits out lined in Table 2 would ensure the successful accomplishment of the UN target maternal mortality rate proposal.

**Table 1:** Shows error analysis for independent variables of SAB, GFR and GDP

Model	Mean Errors	SE Mean
*Log Linear regression (* *1* *)*	*0.008*	*0.171*
*Poisson regression (* *2* *)*	*−216*	*316*

**Table 2:** Optimal values of Ln (SAB) and Ln (GFR) for given Ln (MMR)

Years	MMR Target 140			MMR Target 70		
	Min values of Ln (MMR), changing by 0.11, per a year	Max values of Ln (SAB)	Min values of Ln (GFR)	Min values of Ln (MMR) changing by 0.156	Max values of Ln (SAB)	Min values of Ln (GFR)
2015	6.59	2.95	5.16	6.59	2.95	5.16
2016	6.48	3.01	5.12	6.44	3.04	5.11
2017	6.37	3.08	5.09	6.28	3.13	5.05
2018	6.26	3.14	5.05	6.12	3.22	5.00
2019	6.15	3.21	5.01	5.97	3.31	4.94
2020	6.04	3.27	4.97	5.81	3.40	4.89
2021	5.93	3.33	4.93	5.66	3.49	4.83
2022	5.82	3.40	4.99	5.50	3.58	4.78
2023	5.71	3.46	4.85	5.34	3.67	4.72
2024	5.60	3.52	4.81	5.19	3.76	4.67
2025	5.49	3.59	4.77	5.03	3.85	4.61
2026	5.38	3.65	4.74	4.87	3.94	4.56
2027	5.27	3.71	4.70	4.72	4.03	4.50
2028	5.16	3.78	4.66	4.56	4.12	4.45
2029	5.05	3.84	4.62	4.41	4.22	4.39
2030	4.94	3.91	4.58	4.25	4.33	4.31

The target MMR for 2030 is 140 in the first three columns and 70 in the last three columns

The optimization results presented in Table 2 and Fig. 2 show that if it is desired to reduce MMR to 140 by 2030, we should decrease Ln (MMR) by 0.11 units annually (MMR by 39 units). This will be achieved by simultaneously increasing the value of Ln (SAB) by 0.06 units and reducing Ln (GFR) by 0.04 units. In contrast to achieve MMR of 70, we should decrease Ln (MMR) by 0.156, per year. This requires concurrently increasing Ln (SAB) by 0.09 and decreasing Ln (GFR) by 0.06, per year.

The optimization results presented in Table 3 and Fig. 3 show that to achieve the UN target of 140 by 2030, we should simultaneously increase the value of SAB to 50 and reduce the value of GFR to 97. While to achieve MMR of 70, we should simultaneously increase SAB to 76 and decrease GFR to 75.

**Table 3:** Optimal values of SAB and GFR for a given MMR when GDP) is constant at 1772

Years	MMR Target 140			MMR Target 70		
	Min values of MMR changing by Ln (MMR) = 0.11, per a year	Max values of SAB	Min values of GFR	Min values of MMR changing by Ln (MMR) = 0.156, per a year	Max values of SAB	Min values of GFR
2015	730	19	175	730	19	175
2016	654	20	168	625	21	165
2017	586	22	162	534	23	156
2018	525	23	155	457	25	148
2019	470	25	150	391	27	140
2020	421	26	144	334	30	133
2021	377	28	138	286	33	125
2022	338	30	146	244	36	119
2023	302	32	128	209	39	112
2024	271	34	123	179	43	106
2025	243	36	118	153	47	101
2026	217	39	114	131	52	95
2027	195	41	110	112	57	90
2028	175	44	105	96	62	85
2029	156	47	101	82	68	81
2030	140	50	97	70	76	75

The target MMR for 2030 is 140 in the first three columns and 70 in the last three columns

The target MMR for 2030 is 140.

The target MMR for 2030 is 70.

Figure 4a and b visually show that to achieve minimum MMR we must reduce GRF to its minimum value while increasing SAB to it maximum value. Figure 4b shows sharper reduction in MMR requires deeper increase and decrease in SAB and GFR values respectively.

## Discussion

Reducing the maternal mortality rate has been the main concern of the global health agenda over the last two decades. It has been documented that 74–98% of maternal deaths can be averted even in the lowest income nations [33–38]. According to Chou et al. & Yi et al. improving maternal health and reducing related mortality have been the key concern of the international community as one of the eight Millennium Development Goals (MDG 5) [32, 39]. Maternal mortality is a complex problem requiring complex intervention. It challenges the key performers in resource limited countries to acknowledge the problem and scale up the means and advocated measures to address this problem. The maternal mortality rate in South Sudan is one of the highest in the world. Based on previous studies, authors have identified the Skilled Assistant at Birth (SAB), the General Fertility Rate (GFR) and per capita the Gross Domestic Product (GDP) as the most significant predictors of MMR [10].

The study has deployed mathematical optimization methods and for the first time has developed the optimal lower and upper profile limits for MMR, SAB and GFR. Table 3 provides the yearly optimal values of these variables and can effectively aid the government to achieve the target maternal mortality rate recommended by UN agencies by 2030. The following discussion compares the findings from this research to other similar research, and also seeks to identify the best options for South Sudan to reduce MMR.

### Sab

WHO has defined the term skilled assistant and listed the essential and additional skills required for the expected duties. Although at individual level, skilled assistance can be beneficial, at the population level, evidence is weak. The authors proposed a model which can reduce MMR by 16–33% through primary and secondary prevention of four major complications of pregnancy and delivery. Skilled assistant can reduce mortality rate as well as morbidities. Timely access to quality care and professional staff are also important in the implementation of skilled assistance. In Pakistan, traditional birth attendants are assisting deliveries, but MMR remains high. Training the traditional attendants and then deploying them for assisted deliveries and increasing access to emergency care were found to be very effective [40]. Improved quality of primary care through professional midwifery combined with referral care at hospitals takes less time to reduce MMR significantly. As exemplified by the experiences in some developing countries, the time taken to reduce MMR from 400 to 200 is around 9–12 years, from 200 to 100 is about 7–9 years and for 100 to 50 is 4–8 years [41]. Graham et al., [42] point out that increasing skilled assistance can take time and significant resources and funds. Politics play a critical role in the agenda setting in health affairs; therefore, understanding the priorities of the political agenda in health is vital [43]. Any improvement in the SAB can be influenced by political will. The ingredients of implementing methods to increase SAB in any country are useful for adaption into South Sudan also. The authors have also identified SAB as one of the most influential factors for MMR in South Sudan [10]. Our analysis shows that GFR is the most influential factor in increasing the MMR followed by the SAB and GDP. The South Sudan current statistics shows that only 20% births are attended by skilled assistants [3].

### GFR

In a review of international data, Stover and Ross 2010 [44] found that using contraceptives and family planning reduced MMR by about one million between 1990 and 2005. This was due to the effect of contraceptives on fertility rates, especially in developing countries. Transition from low to high levels of contraceptive use can reduce the country’s MMR by 450 per 100,000 live births. These observations justify the use of contraceptives to reduce GFR in South Sudan. Reduction of unsafe abortion led to significant reduction in MMR in Uruguay between 2001 and 2015, according to the findings of Briozzo 2016 [45]. Thus, abortion as a fertility reduction method among pregnant women is risky. Our study also shows that GFR is the most influential factor in increasing the MMR.

### Other methods to reduce MMR

Mbaruku and Bergström [46] have reported a variety of strategies that resulted in reducing MMR from 933 to 186 per 100,000 live births during 1984–91 in Tanzania. Low cost strategies combined with local problem solving methods show that it is possible for developing countries, like South Sudan, to achieve significant reduction in MMR level.

Lassi and Bhutta (2015) in a review stressed community-based integrated packages of care for both mothers and new-born babies to help reduce MMR along-with neonatal mortality [47].

### Best option

Clear evidence on reduction of MMR by the use of contraceptives was provided in the review of data on 172 countries by Ahmed et al. [48]. Using predictive models, they estimated that 342,203 women died of maternal causes in 2008. Contraceptive use prevented death of another 272,040 (44%) deaths. If unmet needs of contraceptive use were satisfied, another 104,000 maternal deaths could be prevented every year. The basic model of the authors for estimation of MMR is.

$$ \mathrm{Log}\ \left(\mathrm{PMDFi}\right)={\upbeta}_0+{\upbeta}_1\log\ \left({\mathrm{GDP}}_{\mathrm{i}}\right)+{\upbeta}_2\log\ \left({\mathrm{GFR}}_{\mathrm{i}}\right)+{\upbeta}_3{\mathrm{SAB}}_{\mathrm{i}}+{\upalpha^{\mathrm{c}}}_{\mathrm{j}\left[\mathrm{i}\right]}+{\upalpha}^{\mathrm{R}}{\mathrm{k}}_{\left[\mathrm{i}\right]}+\log\ \left(1-{\mathrm{a}}_{\mathrm{i}}\right)+{\upvarepsilon}_{\mathrm{i}} $$

This is similar to the model used in this paper.

In the above equation, PMDF is the proportion of maternal deaths among all deaths of reproductive-age women (15–49 years) in year i, country j, and geographical region k. While α^c^ and α^R^ are random intercepts for country j in geographical region k, respectively, a_i_ is the proportion of death due to AIDS among the women of reproductive age, and ε_i_ is the error term. The GDP was adjusted according to purchasing power parity in 2005. But they have modified the equation for predicting MMR for contraceptive use.

In conclusion, the outlined research in other countries shows that increase in SAB and decrease in GFR can reduce MMR. These are consistent with the findings of this paper.

Based on the above discussion it appears that initiatives to increase SAB and reduce GFR are vitally important for reducing MMR. However, in a short run, reducing GFR is relatively easier than increasing SAB.

### Limitations

#### Factors impacting MMR

This paper investigates the impact of SAB, GFR and GDP on maternal mortality rate in South Sudan. However, as highlighted in the introduction, factors impacting the maternal mortality rate include socio-economic factors, macro-economic factors and physiological factors [1]. Lack of access to health care facilities is also a major factor due to lack of roads and transportation system [7, 10]. More than 50% of the population walks three miles or more to the nearest primary health care unit. All of factors affect the total health care system and in particular high maternal mortality rate problem. Kruk et al. have investigated the impact of the community perceptions on the quality of care provided by the local health system on pregnant woman’s decision to deliver in a clinic. They have suggested that improving the quality of care at first level clinics may assist the efforts to increase facility delivery in sub-Saharan Africa [2].

### Data

The proposed Ln-linear regression model deployed in this paper and the constraints on SAB and GFR used to develop the profiles are based on 30 years of manually recorded data obtained from the sources listed under “data collection”. The authors acknowledge that some of these data may be an estimate rather than the true values. Although this may result in underestimate/overestimate of the profiles, it is unlikely to impact on the validity of the analyses. Furthermore, our results on the impact of GFR, SAB and GDP on MMR in South Sudan are aligned with that of other researchers (as highlighted in the discussion section).

## Conclusions

This study for the first time has deployed optimisation procedures to develop yearly lower and upper profile limits for maternal mortality rate (MMR), targeting the UN recommended lower and upper MMR levels by 2030. The MMR profile limits have been accompanied in by the profile limits for optimal yearly values of SAB and GFR levels. Studies on predictors of logarithmic multi-regression models provided distinct evidence that increasing Skilled Attendant at Birth (SAB) and decreasing General Fertility Rate (GFR) while leaving the Gross Domestic Product (GDP) constant at 1772, can reduce the Maternal Mortality Rate in South Sudan by 2030 to the limits proposed by the UN agencies (WHO, USAID, UNICEF & World Bank, 2015) and beyond. The statistical analysis shows that increasing SAB by 1.22% per year would decrease MMR by 1.4%. [95% CI (0.4–5%)] while decreasing GFR by 1.22% per year would decrease MMR by 1.8% [95% CI (0.5% - 6.26], when the GDP is held constant.

The comparison of the findings of this study to other similar studies suggests that reducing GFR is more effective and achievable than increasing SAB when aiming to reduce MMR.

The optimal profile limits provide a quantitative guide-line for the government and partners in terms of yearly SAB and GFR target in order to reduce MMR to the level recommended by the UN. The outcomes of this study can effectively aid authorities to make informed evidence-based intervention decisions on resources allocation to reduce the MMR.

## Abbreviations

AIDs: Acquired Immune Deficiency Syndrome (AIDs)
GDP: Gross Domestic Product (GDP)
GFR: General Fertility Rate (GFR)
HIV^+^: Human Immunodeficiency Virus
Inc.: increment (See Fig. 1)
Ln: Logarithmic of natural number
MATLAB: Matrix Laboratory is multi-paradigm numerical computing environment and fourth- generation programming language
MDG: Millennium Development Goals
MDs: Maternal Deaths
Minitab: Statistical analysis software
MMR: Maternal Mortality Rate
R: Multi-paradigm numerical computing environment and generation of computer programming language
Red: reduction (See Fig. 1)
SAB: Skilled Attendance at Births
SSA: Sub-Saharan Africa (a region in Africa)
UNICEF: United Nations International Children’s Emergency Fund
USAID: United States Agency for International Development
USD: United States Dollar
WHO: World Health Organisation
